# Fraction of high-grade cervical intraepithelial lesions attributable to genotypes targeted by a nonavalent HPV vaccine in Galicia, Spain

**DOI:** 10.1186/s12985-017-0879-1

**Published:** 2017-11-06

**Authors:** S. Perez, A. Iñarrea, R. Pérez-Tanoira, M. Gil, E. López-Díez, O. Valenzuela, M. Porto, L. Alberte-Lista, M. A. Peteiro-Cancelo, A. Treinta, R. Carballo, M. C. Reboredo, M. E. Alvarez-Argüelles, M. J. Purriños

**Affiliations:** 1 Microbiology Department, Institute of Biomedical Research of Vigo, University Hospital of Vigo, Vigo, Spain; 2 Gynecology Department, University Hospital of Vigo, Vigo, Spain; 3 Internal Medicine Department, Hospital Fundación Jiménez Díaz, Madrid, Spain; 4 Urology Department, University Hospital of Vigo, Vigo, Spain; 5 Pathology Department, University Hospital of Vigo, Vigo, Spain; 6 Virology Department, Central University Hospital of Asturias, Oviedo, Spain; 7 Health and Epidemiology Department. Innovation and management of public health. Consellería de Sanidade, Xunta de Galicia, Santiago de Compostela, A Coruña Spain

**Keywords:** Nonavalent vaccine, HPV, Potential impact, Cervical lesion, Additional impact

## Abstract

**Background:**

Human papillomavirus (HPV) bivalent and quadrivalent vaccines have been widely implemented in worldwide organized immunization programs. A nonavalent HPV vaccine is now available in several countries. The objective was to describe the fraction of squamous non-invasive high-grade cervical intraepithelial lesions attributable to genotypes targeted by bi-quadrivalent vaccines and by nonavalent vaccine according to age and diagnosis in women living in the city of Vigo (Galicia, Spain).

**Methods:**

Cervical scrapings (2009–2014) of women with histological diagnosis of cervical intraepithelial neoplasia grade 2 (CIN2, *n* = 145) and grade 3-carcinoma in situ (CIN3-CIS, *n* = 244) were tested with Linear Array HPV Genotyping test (Roche diagnostics, Mannheim, Germany). Hierarchical estimation of the fraction attributable to HPV 16/18 or HPV 31/33/45/52/58 detected alone or in combination was calculated. Absolute additional fraction attributable to genotypes targeted by nonavalent vaccine compared to genotypes targeted by bi-quadrivalent vaccines was calculated as the increment of attributable cases with respect to all studied cases. Age group 1, 2 and 3 included women 18 to 34, 35–44 and ≥45 years old, respectively. EPIDAT 3.1 was used.

**Results:**

Fraction attributable to genotypes targeted by bi-quadrivalent vaccines was 59% CIN2 vs. 69% CIN3-CIS (*p* < 0.001). It was 63/51/50% of CIN2 and 78/66/45% of CIN3-CIS in age group 1, 2, 3, respectively. Fraction attributable to genotypes targeted by nonavalent vaccine was 86% CIN2 and 86% CIN3-CIS. It was 87/91/75% of CIN2 and 90/86/76% of CIN3-CIS in age group 1, 2, 3, respectively. Fraction attributable to genotypes targeted by these vaccines tended to decrease as age increased (*p*-trend <0.05). Globally, absolute additional attributable fraction was 16%, 26% and 29% in age group 1, 2 and 3, respectively (*p* < 0.005).

**Conclusions:**

Absolute additional fraction of CIN2 and CIN3-CIS attributable to genotypes targeted by nonavalent vaccine was observed in women of any age, especially in those over 35 years old.

## Background

Human papillomavirus (HPV) vaccines have demonstrated their preventive potential for different HPV-related diseases [1, 2]. Invasive cervical cancer (ICC), the fourth most common women cancer worldwide [3], is caused by high risk (HR) HPV genotypes. Around 80 countries have implemented HPV immunization programmes for cervical cancer prevention since 2006 to 2014 [4]. Their impact will depend on vaccination coverage and vaccine efficacy. Vaccination coverage is uneven, higher in high and upper-middle income countries. Vaccine efficacy is considered to be very high for the targeted genotypes. “First generation” vaccines are the bivalent vaccine (2-valent, Cervarix®, GlaxoSmithKline) which targets HPV 16/18 and the quadrivalent vaccine (4-valent, GARDASIL®/Silgard®, Merck&Co) which targets HPV 6/11/16/18. “Second generation” vaccine is the nonavalent vaccine (9-valent, GARDASIL 9®, Merck&Co) which targets HPV 6/11/16/18/31/33/45/52/58. It was licensed in December 2014 and is currently undergoing World Health Organization (WHO) review for prequalification. In consequence, most European countries recommend and/or fund 2-valent or 4-valent vaccines and a few recommend 9-valent vaccine [5].

No HPV vaccine protects against all HR HPV genotypes responsible for ICC. HPV 16 and HPV 18 cause the majority -around 70%- of ICC. HPV genotypes targeted by 9-valent vaccine are responsible for approximately 90% of ICC. The process for making a decision about introducing HPV vaccine into an immunization programme or about changing to a second generation vaccine has to be systematic and transparent [6, 7]. Other coordinated strategies should be carried out at the same time as vaccination: On one side health and sexual education and on the other side suitable screening and treatment of cervical lesions. It has been previously described age-specific distribution of some HPV genotypes in cervical neoplasia and ICC [8–10] and they might influence the cost-effectiveness of vaccination with 9-valent vaccine and the screening of vaccinated populations.

The objective of this study was to describe the fraction of squamous non-invasive high-grade cervical intraepithelial lesions attributable to HPV genotypes targeted by a 9-valent vaccine and by 2/4-valent vaccines according to age and diagnosis in women living in the city of Vigo (Galicia, Spain).

## Methods

### Patients

Women with histological diagnosis of cervical intraepithelial neoplasia grade 2 (CIN2, *n* = 123) or grade 3-*carcinoma in situ* (CIN3-CIS, *n* = 193) were prospectively recruited between the years 2011–2014 in the University Hospital of Vigo, Spain. Women with CIN2 (*n* = 22) and CIN3-CIS (*n* = 51) lesions histologically diagnosed from 2009 to 2010 in the same hospital were also included in this study. Characteristics of these 2009–2010 cases were previously described in a retrospective study [11]. Age at first worst histological diagnosis was reported. A woman was counted multiple times if developed a second lesion after treatment. The patients were not included in an HPV vaccination programme of preadolescent girls.

### Histological diagnoses

Cervical biopsy specimens (colposcopy, conization or hysterectomy) were studied for histological diagnosis. CIN2–3 included CIN2 and CIN3-CIS.

### Genotype specific HPV prevalence

Endocervical scrapings were collected for HPV detection in TE buffer pH 8.0 Molecular Biology grade (AppliChem GmbH, Darmstadt, Germany). They were taken at CIN2–3 diagnosis time or within the previous 4 months. QIAamp MinElute Media Kit (Qiagen, Hilden, Germany) and Linear Array HPV Genotyping Test (Roche Diagnostics, Mannheim, Germany) were respectively used for DNA extraction and HPV genotyping. Eight cervical biopsies of CIN2 and 22 biopsies of CIN3 were used for the retrospective study as previously described [11]. In case of HPV 33, 35 and/or 58 infection, specific PCR for HPV 52 detection was performed following a previous publication [12].

HPV genotypes were classified attending the International Agency of Research on Cancer (IARC) classification [13] and their inclusion in current vaccines: (a) HR genotypes targeted by 2/4-valent vaccines (HPV 16 and 18), (b) HR genotypes targeted by 9-valent vaccine, other than HPV 16/18 (HPV 31, 33, 45, 52 and 58), (c) HR genotypes not targeted by current vaccines (HPV 35, 39, 51, 56 and 59), (d) genotypes of probable or possible HR (HPV 26, 53, 66, 67, 68, 69, 70, 73 and 82). Low risk genotypes were not considered.

Three approaches were used to estimate the attribution of individual genotypes to cervical lesions [14]. Two HPV estimate methods were considered to converge when difference was ≤5%.

#### Minimum estimate

Was calculated by including in the numerator the number of lesions with each genotype detected in single infection. Lesions included in the denominator were all single genotype lesions.

For the rest of estimations, all lesions (i.e., both HPV positive and HPV negative) were included in the denominator, as the HPV-negative lesions may have been caused by a non-tested type. In the numerator, both single and multiple genotype infections were considered but they used different attribution methods for multiple genotype infections:

#### Proportional attribution estimate

In case of multiple genotype lesion, it included in the numerator a fractional allocation for each individual genotype. It was based on the relative number of instances in which each genotype was observed as a single infection in this study. For example, if there were 3 HPV 16/31 infected lesions, and if there were 10 lesions with HPV 16 single infection and 1 lesion with HPV 31 single infection, then [3 × 10/ (10+ 1)] or 2.7 of these 3 multiple type infected lesions would be attributed to HPV 16 and [3 × 1/ (10 + 1] or 0.3 would be attributed to HPV 31.

#### Hierarchical attribution estimate

Cervical lesions with multiple infection were attributed to the detected genotype belonging to the HPV group most commonly detected in ICC. For example, a lesion was attributed to HPV 31/33/45/52/58 (i.e., the additional HR genotypes included by 9-valent vaccine), only if there were not detected HPV 16 and/or HPV 18 (i.e., the HR genotypes included by 2/4-valent vaccines). For example, a lesion was attributed to HPV 35/39/51/56/59 (i.e., the HR genotypes not targeted by current vaccines) only if there were not detected HPV 16/18/31/33/45/52/58.

### Data analysis

Absolute additional fraction of cervical lesions attributable to genotypes targeted by 9-valent vaccine compared to 2/4-valent vaccines was calculated as the increment of attributable cases with respect to all studied cases. Three age groups were considered: Group 1 (18–34 years), 2 (35–44 years) and 3 (≥45 years).

For means, standard deviation (SD) and 95% confidence interval (95%CI) were calculated. Quantitative values were compared by Chi-square test. A two-sided *p*-value <0.05 or *p*-trend value <0.05 were considered statistically significant (EPIDAT software version 3.1) [15].

### Ethics statement

Ethics Committee of Clinical Investigation of Galicia approved this study (CEIC reference number 2008/190). Women signed a consent form before their participation.

## Results

### Population characteristics

In this study, 389 cases (386 women, 18–75 years old) were included. Global mean age was 35.3 years (SD 10.2, 95%CI: 34.3–36.3). Mean age of CIN2 cases was 33.5 years (SD 10.2, 95%CI: 31.8–35.2). Mean age of CIN3-CIS cases was 36.4 years (SD 10.2, 95%CI: 35.1–37.7) (Table 1).

**Table 1:** Distribution of women included in the study by age and cervical lesion grade

Cervical lesion	Age group (n)			Total
	18–34 years old	35–44 years old	≥45 years old	
CIN2	90	35	20	145
CIN3-CIS	129	77	38	244
TOTAL	219	112	58	389

HR HPV was detected in 386/389 lesions. Single genotype infection was detected in 66 CIN2 and 135 CIN3-CIS lesions. Multiple genotype infection was detected in 77 CIN2 and 108 CIN3-CIS lesions.

### Prevalence of vaccine and non-vaccine HR HPV genotypes in single genotype lesions

**Fig. 1:**
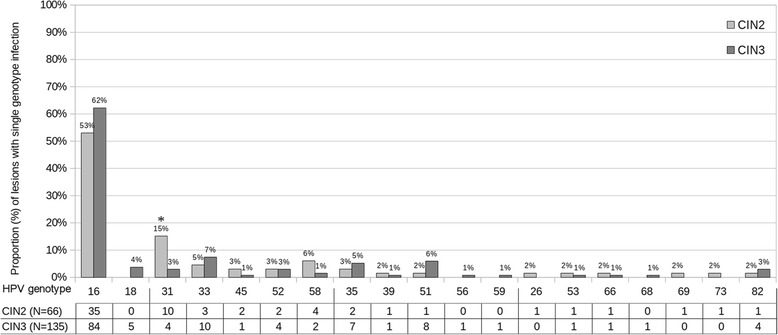
Prevalence of HPV genotypes in single genotype infections. *p < 0.01 for the comparison of individual genotype prevalence between CIN2 and CIN3-CIS

Prevalence of individual HPV genotypes is shown in Fig. 1 considering only single genotype infections (*n* = 201). The most frequently detected genotypes were HPV 16 among 2/4-valent vaccine genotypes, HPV 31/33 among 9-valent vaccine genotypes other than HPV 16/18, HPV 35/51 among HR non vaccine genotypes and HPV 82 among probable/possible HR genotypes. A linear trend was found for HPV 16 prevalence (n/N, %) in age group 1 (63/94, 67.0), 2 (36/63, 57.1) and 3 (20/44, 45.4) (*p* = 0.01). Prevalence of HPV 31 was 15.1% (10/66) in CIN2 vs 3.0% (4/135) in CIN3-CIS (*p* = 0.003).

### Attributable fraction according to different estimate methods

**Fig. 2:**
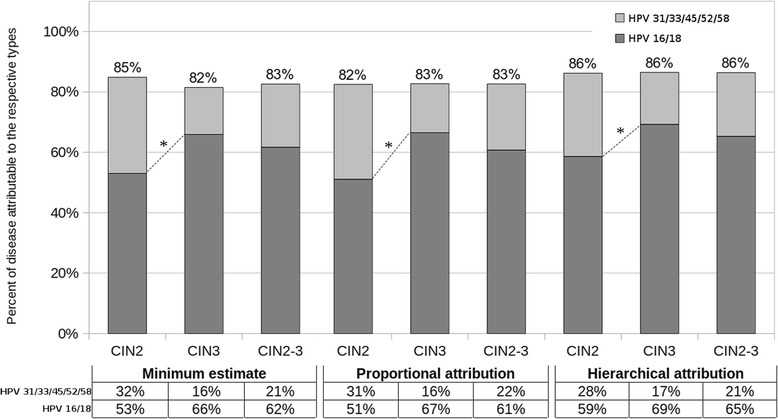
Fraction of cervical lesions attributable to HPV genotypes targeted by bi-quadrivalent vaccines (HPV 16/18) or nonavalent vaccine (HPV 16/18 and HPV 31/33/45/52/58). *p < 0.001 for the comparison of each HPV group prevalence between CIN2 and CIN3-CIS

Figure 2 shows the percent of disease attributable to HR genotypes included in the 2/4-valent vaccines and in the 9-valent vaccine calculated by different estimate methods considering single and multiple genotype infections (*n* = 386). Data of 2/4-valent vaccine genotypes converged for all estimate methods except for hierarchical attribution and crude prevalence in CIN2 (6% difference with respect to minimum estimate)*.* Fraction attributable to 2/4-valent vaccine genotypes was 53.0% (35/66) of CIN2 vs. 65.9% (89/135) of CIN3-CIS (*p* < 0.001) (minimum estimate) and 58.6% (85/145) of CIN2 vs. 69.3% (169/244) of CIN3-CIS (*p* < 0.001) (hierarchical attribution). Data of 9-valent vaccine genotypes converged for all estimate methods. Fraction attributable to 9-valent vaccine genotypes was 84.8% (56/66) of CIN2 vs. 81.5% (110/135) of CIN3-CIS (minimum estimate) and 86.2% (125/145) of CIN2 vs. 86.5% (211/244) of CIN3-CIS (hierarchical attribution).

### Attributable fraction according to age

Age specific attributable fraction according to lesion grade is shown in Table 2. Fraction of CIN2–3 attributable to 2/4-valent vaccine genotypes tended to decrease with increasing age (*p*-trend <0.05). Considering hierarchical attribution, fraction of CIN2–3 attributable to 9-valent vaccine genotypes also tended to decrease with increasing age (*p*-trend <0.05).

**Table 2:** Age specific attribution of precancerous cervical lesions to vaccine HPV genotypes attending three estimate methods

		CIN2			CIN3-CIS			CIN2–3		
Method	Women age (years)	HPV 16/18 (%)	HPV 31/33/45/52/58 (%)	*p*	HPV 16/18 (%)	HPV 31/33/45/52/58 (%)	*p*	HPV 16/18 (%)	HPV 31/33/45/52/58 (%)	*p*
Minimum estimate	18–34	64	25		71	14	*	69	17	*
	35–44	52	39		68	13		62	22	*
	≥45	33	33		52	24		45	27	*
	*p-trend*							*		
Proportional attribution	18–34	53	29	*	74	13	*	66	19	*
	35–44	48	41	*	64	16	*	59	24	*
	≥45	48	26		44	28	*	46	27	*
	*p-trend*							*	*	
Hierarchical attribution	18–34	63	23	*	78	12	*	72	16	*
	35–44	51	40	*	66	19	*	62	26	*
	≥45	50	25		45	32	*	47	29	*
	*p-trend*							*	*	

*p-trend < 0.05 for each HPV group prevalence decrease with increasing age

*p < 0.05 for absolute additional fraction of cervical lesions attributable to HPV genotypes targeted by nonavalent vaccine compared to the fraction attributable to those targeted by bi/quadrivalent vaccines

### Absolute additional fraction attributable to nonavalent vaccine genotypes

For 9-valent vaccine genotypes, absolute additional attributable fraction of CIN2–3 was observed for all age groups (*p* < 0.05) (Table 2). Additional 16.4% (36/219), 25.9% (29/112) and 29.3% (17/58) of CIN2–3 were attributable to 9-valent vaccine genotypes in age group 1, 2 and 3, respectively (*p* < 0.005), in comparison with 2/4-valent vaccine genotypes.

## Discussion

In this work, almost four hundred squamous precancerous cervical lesions were studied. The first objective was to estimate local age-specific fraction of squamous non-invasive high-grade cervical intraepithelial lesions attributable to HPV genotypes targeted by current vaccines. The second objective was to compare additional fraction attributable to 9-valent vaccine genotypes in comparison with 2/4-valent vaccine genotypes.

In this paper, it was considered that each HPV vaccine would have the potential to prevent those lesions caused by the targeted genotypes. These genotypes might be detected in single or multiple genotype infection. When a multiple genotype infection was found it was not easy to make a correct attribution of each genotype to the lesion. Three methods were used to determine the attribution of HR genotypes. These approaches yielded almost similar results. Minimum estimate might be considered the most reliable method because data are directly obtained without mathematical calculations. Hierarchical and proportional attribution methods were very useful as multiple genotype infections could be included in the calculations. They yielded valuable results of twice as many samples as the minimum estimate method. Hierarchical attribution might have a strong clinical signification. Hierarchical attribution considered that the HR genotypes targeted by the 2/4-valent vaccines were the most oncogenic genotypes, followed by those targeted by the 9-valent vaccine and those not included in any vaccine. Hierarchical attribution indicated the upper limit of attributable fraction. Proportional attribution was the most complicated method. It could be the most influenced by local prevalence of individual genotypes in CIN2–3.

Bi-quadrivalent vaccine genotype attributable fraction was higher for CIN3-CIS than for CIN2 according to HPV 16 and HPV 18 prevalence. Nonavalent vaccine genotype attributable fraction was similar for CIN2 and CIN3-CIS. It could reach values of 86% CIN2–3, as described by Hartwig et al. (82%), Joste et al. (70% CIN2, 84% CIN3) and Riethmuller et al. (90%) [2, 9, 16]. Thus, attending to the attributable fraction, changing 2/4-valent vaccines for 9-valent vaccine in the vaccination of preadolescent girls could increase at most 16–29% CIN2–3 prevention as previously described [2, 9, 16].

The lowest oncogenic HPV genotypes were found mainly in CIN2–3 lesions diagnosed in the oldest women. This influenced the analysis of the vaccine genotype attributable fraction in each specific age group. First, vaccine genotype attributable fraction decreased as age increased. Secondly, the greatest absolute additional protective effect would be observed when vaccinated preadolescent women were more than 35 years old.

Half cases were infected by only one genotype. These single infections could be useful for testing if global results obtained after mathematical attribution of HPV genotypes were reasonable. Among these single genotype lesions, HPV 16 was more prevalent in the youngest than in the oldest women, as previously reported [9, 11]; HPV 31 was less prevalent in CIN3-CIS than in CIN2. These data are in concordance with the potential vaccine impact discussed above. These vaccines seem to have more potential impact on the youngest women, among whom HPV 16 is more prevalent. Bi-quadrivalent vaccine genotype attributable fraction seems to be higher for CIN3-CIS than for CIN2. This could be related with the lower prevalence of at least HPV 31 in CIN3-CIS.

The introduction of 2/4-valent vaccines has demonstrated to reduce cervical abnormalities, genital warts and HPV prevalence as well as the appearance of herd immunity after immunization programmes [17–20]. There are new current challenges like to decide the screening of vaccinated women, the change to second generation vaccines or the introduction of vaccines for anal cancer prevention. Data from international studies as well as local data could support these decisions.

Limitations: Quadrivalent and bivalent vaccine genotype attributable fraction of lesions was considered to be the same. The possible oncogenic effect of HPV 6/11 (low risk genotypes included in the quadrivalent vaccine) has not been taken into account. Cross protection against other related genotypes was not considered. Nonavalent vaccine antigens might probably produce a much stronger and longer lasting immunogenic response than cross protection. It would not be simple to establish whether the immunity directly induced by a vaccine and the cross-protection should be quantified in the same way. In this study, influence of vaccination coverage and vaccine efficacy were not taken into account. Current Spanish vaccine coverage is high [21], exceeding the threshold for optimum cost-effectiveness (70%) [22]. Suboptimal vaccination coverage or suboptimal vaccine efficacy might reduce the potential protection calculated in this study. Women were not included in an HPV vaccination National programme but they could have received adult non-funded vaccination. The effect of adult vaccination was not considered.

## Conclusions

In comparison with bivalent and quadrivalent HPV vaccine, additional fraction of CIN2 and CIN3-CIS attributable to genotypes targeted by nonavalent HPV vaccine was observed in women of any age, especially on women more than 35 years old. Age-specific potential impact of nonavalent HPV vaccine should be taken into account in cost-effectiveness evaluations of HPV immunization programmes and in the organization of screening of vaccinated populations.

## Abbreviations

2/4-valent: Bivalent or quadrivalent
2-valent: Bivalent
4-valent: Quadrivalent
9-valent: Nonavalent
CEIC: Ethics Committee of Clinical Investigation of Galicia
CI: Confidence interval
CIN2: Cervical intraepithelial neoplasia grade 2
CIN2–3: CIN2 and CIN3-CIS
CIN3-CIS: Cervical intraepithelial neoplasia grade 3-carcinoma in situHPV: Human papillomavirus
HR: High risk
IARC: International Agency of Research on Cancer
ICC: Invasive cervical cancer
SD: Standard deviation
WHO: World Health Organization
