# Correction to: Fraction of high-grade cervical intraepithelial lesions attributable to genotypes targeted by a nonavalent HPV vaccine in Galicia, Spain

**DOI:** 10.1186/s12985-018-0953-3

**Published:** 2018-03-06

**Authors:** S. Perez, A. Iñarrea, R. Pérez-Tanoira, M. Gil, E. López-Díez, O. Valenzuela, M. Porto, L. Alberte-Lista, M. A. Peteiro-Cancelo, A. Treinta, R. Carballo, M. C. Reboredo, M. E. Alvarez-Argüelles, M. J. Purriños

**Affiliations:** 1 Microbiology Department, Institute of Biomedical Research of Vigo, University Hospital of Vigo, Vigo, Spain; 2 Gynecology Department, University Hospital of Vigo, Vigo, Spain; 3 Internal Medicine Department, Hospital Fundación Jiménez Díaz, Madrid, Spain; 4 Urology Department, University Hospital of Vigo, Vigo, Spain; 5 Pathology Department, University Hospital of Vigo, Vigo, Spain; 6 Virology Department, Central University Hospital of Asturias, Oviedo, Spain; 7 Health and Epidemiology Department, Innovation and management of public health, Consellería de Sanidade, Xunta de Galicia, Santiago de Compostela, A Coruña Spain; 8 Universidade de Vigo, Pontevedra, Spain

## Correction

After publication of the article [1], it was brought to our attention that the author E. López-Díez is missing their second affiliation. The author would also like to indicate an affiliation to “Universidade de Vigo, Pontevedra, Spain”.
